# Optical Characterization of Thin Films by Surface Plasmon Resonance Spectroscopy Using an Acousto-Optic Tunable Filter

**DOI:** 10.3390/ma16051820

**Published:** 2023-02-22

**Authors:** Ildus Sh. Khasanov, Boris A. Knyazev, Sergey A. Lobastov, Alexander V. Anisimov, Pavel A. Nikitin, Oleg E. Kameshkov

**Affiliations:** 1 Scientific and Technological Centre of Unique Instrumentation RAS, 117342 Moscow, Russia; 2 Budker Institute of Nuclear Physics SB RAS, 630090 Novosibirsk, Russia

**Keywords:** acousto-optic, tunable filter, surface plasmon resonance, surface electromagnetic wave spectroscopy, thin film thickness, wavelength and angular interrogation, refractive index sensing

## Abstract

The paper presents the application of the acousto-optic tunable filter (AOTF) in surface plasmon resonance (SPR) spectroscopy to measure the optical thickness of thin dielectric coatings. The technique presented uses combined angular and spectral interrogation modes to obtain the reflection coefficient under the condition of SPR. Surface electromagnetic waves were excited in the Kretschmann geometry, with the AOTF serving as a monochromator and polarizer of light from a white broadband radiation source. The experiments highlighted the high sensitivity of the method and the lower amount of noise in the resonance curves compared with the laser light source. This optical technique can be implemented for nondestructive testing in the production of thin films in not only the visible, but also the infrared and terahertz ranges.

## 1. Introduction

Recent advances in nanofabrication techniques have facilitated the development of new materials such as metasurfaces [1], gradient-index films [2], and so on. Such nanomaterials have sparked renewed interest in plasmonics [3], a field of research that aims to control light–matter interactions at the nanoscale at metal–dielectric interfaces. For the same reason, the methods for controlling the deposition and characterization of thin films for accurate measurement of their plasmonic performance have been receiving increased interest [4].

Surface plasmon resonance (SPR) is one of the most sensitive techniques for the optical characterization of thin layers whose thickness is much less than the wavelength of the probing light [5,6]. SPR is a well-established biosensing technique for environmental and medical applications for the detection and characterization of various analytes [7,8,9,10]. Like other reflectometry techniques, the SPR method consists of the measurement of the reflection coefficient *R* [6]. *R* depends on the wavelength, angle of incidence *θ*, film thickness, and optical constants of the multilayer structure under study. SPR was observed not only in the visible, but also in the infrared [11,12] and terahertz ranges [13,14]. Usually, the wavelength interrogation mode, i.e., SPR spectroscopy, is used in combination with optical fibers [15], thus the angular interrogation mode cannot be applied. Application of the acousto-optic tunable filter (AOTF) allows combining angular and spectral modes.

Since its development in 1969 [16], the AOTF has found applications in many optical research methods, such as spectroscopy [17], profilometry [18], microscopy [19], endoscopy [20], stereoscopy [21], colorimetry [22], holography [23,24], and so on, thanks to its high tuning speed, narrow spectral width, low distortion of the passing collimated beam [25], compactness, absence of moving mechanical parts, and electronic control. The AOTF is suitable for any research that requires spectral image processing [26]. The AOTF enables a dynamic increase in the imaging contrast of objects under study [27], attenuation of the laser radiation [28], and so on. The AOTF relies on the interaction of light with sound [29]; а piezoelectric transducer—when radiofrequency is applied—generates an acoustic wave, which creates in the birefringent medium periodic regions of compression and decompression with different refractive indices. The traveling light wave diffracts on these areas like on a diffraction grating, the period of which can be adjusted through the sound frequency for dynamic spectral selection. The diffraction efficiency of the AO interaction in new AO devices has improved significantly [30,31,32], and the number of applicable materials with optical anisotropy has increased [33,34]. Owing to the choice of new AO media (including liquid ones [35]), the spectral range has been extended from the ultraviolet and visible [36] to the infrared [37,38] and terahertz [39,40] ranges.

The narrow spectral width (which can theoretically be as low as 2.5 Å for λ = 630 nm [41]) from the emitted spectrum of the radiation source and the high degree of polarization is an ideal combination for surface plasmon resonance (SPR) spectroscopy, as this effect is observed in a monochromatic *p*-polarized radiation [42]. In this case, using a radiation with a short coherence length, as in the case of an AOTF-filtered white light source, may be preferable to the use of coherent laser radiation. Although the SPR phenomenon has a quantum nature as it occurs when light tunnels through a thin metal film, we can accurately calculate the reflection coefficient *R* within the transfer matrix formalism, similarly to its use in classical optics, to describe interference in thin films. However, it is essential to note that the thickness of the metallic film supporting SPR should not exceed half the wavelength and is usually much smaller than it. So, the coherence length of light does not affect the calculation of *R* because it does not exceed the thickness of the film. Therefore, SPR can be similarly observed with the coherent laser light or incoherent quasimonochromatic light. The latter is more favorable owing to the suppression of parasitic interference in lenses, as well as of spurious diffraction such as speckles from scattering on surface roughness, dust particles, and other minor inhomogeneities of the films. Therefore, the use of the white light filtered by the AOTF for the creation of monochromatic incoherent radiation makes it possible to obtain resonance curves containing less noise compared with the laser radiation.

The AOTF was first applied to SPR measurements in [43,44] in the fixed-angle wavelength interrogation mode. It has been shown that, in the Kretschmann configuration, it is possible to increase the sensitivity of the sensor by combining angular and spectral modulation via selection of the optimal wavelength and angle [45]. Recently, we have developed an algorithm [46] that allows using the full range of scanning angles and wavelengths to increase the accuracy of SPR measurements. Our goal is to show the effectiveness and prospects of our approach to popularize the application of the AOTF in SPR spectroscopy.

## 2. Materials and Methods

To experimentally demonstrate the AOTF operation in SPR spectroscopy in the combined spectral and angular interrogation mode, we use an AO cell made of paratellurite at the STC UI RAS. The main parameters of the AO cell are described in [36] (TeO_2_ material, γ = 7°, diffraction mode e → o, crystal length of 25 mm, 10 mm × 8 mm light aperture, and spectral range of 450–900 and 900–1700 nm). The chromatic shift of the AO cell is compensated for with a heavy flint (TF-1) wedge and is equal to approximately 5’.

### 2.1. Optical Setup

**Figure 1:**
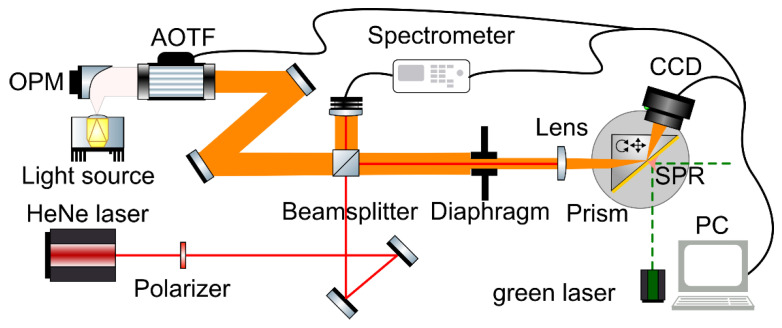
The optical scheme of the AOTF-based spectroscopy. The light source is the ISTEQ XWS-65 laser-pumped plasma broad white light radiation source collimated with the OPM (off-axis parabolic mirror) Thorlabs MPD399-M03 with a diameter of Ø3” and reflected focal length = 9”; HeNe laser is a helium-neon laser LGN-301 with an emission wavelength of 632.8 nm; Beamsplitter is a non-polarizing 50/50 cube with a 25 mm facet; Diaphragm is an 8 mm aperture for beam collimation; Lens is a Thorlabs achromatic lens Ø1” with a spectral range of 400–700 nm and focal distance of 50 mm; Polarizer is a visible range polarizer; Spectrometer is a tunable grating spectrometer Avesta ASP-150 with Hamamatsu S8378-1024Q CCD array and a spectral resolution of 0.06 nm; Prism is a right-angle prism (produced at STC UI RAS) with base dimensions of 22 mm and height of 11 mm, made of LC-7 glass with a metal (silver, Ag) film and dielectric (silicon dioxide, SO_2_) coating and placed on a rotating miniplatform Standa 7R7 mounted on a translation stage Standa 7T173 and vertical translation stage Standa 7VT188-20; CCD is a GigE monochrome industrial camera Imaging Source DMK 23G445 with no IR filter, with a resolution of 1280 × 960 pixels and sensitivity of down to 0.015 lx; PC is a personal computer with the control software written in Python 3 and AutoIt 3.

The optical scheme of the experimental setup is shown in Figure 1.

To create a uniform collimated light without chromatism, we use a parabolic off-axis mirror with the fiber optic output of the white light source in the focus. The AO cell control driver enables wavelength selection. A collimated *p*-polarized radiation beam deflected by the AO cell is directed to the object under study; that is, a prism with a thin film coating on its base. The right-angle prism was made of LC-7 glass at the optical division of the STC UI RAS. LC-7 is a Russian National State Standard (GOST) light crown glass with a refractive index *n* of about 1.48 and dispersion coefficient ν_e_ = 66.17. With the help of a beam-splitting cube, part of this radiation is deflected for analysis to the spectrometer. This enables determination of the wavelength without cell pre-calibration, as well as control of possible temperature deviations during the measurements [47]. We found that the AOTF characteristics remained stable during the experiment. The measurements are performed at a fixed angle of the rotating miniplatform, which is preselected such that the resonance dip is present in all images (as the resonance dip shifts monotonically towards lower angles with increasing wavelength). The angular interrogation is provided by the concentration of the beam by a focusing lens. The focal spot is at the base of the prism, i.e., on the thin film. The accuracy of the lens focusing is controlled by means of the camera via choice of the lens position corresponding to the minimum light spot visible from the back side of the prism. The focus spot does not exceed 0.5 mm. The cleanliness and homogeneity of the measured region of the thin film are also controlled by means of the HeNe laser, as a coherent radiation is more sensitive to roughness, defects, and contamination of the thin film surface. We are able to select a region of the thin film using micromechanical horizontal and vertical translators.

### 2.2. Preparation of Samples

The object of the study was a thin film of silver (Ag) with a thickness of 55 nm. The coating was performed with a vacuum unit for ion-beam sputtering of optical coatings (the Aspira sputtering unit, OOO “FLAVT”, Serpukhov, Russia). 

At first, a set of silver-coated prisms was made, and then each prism was separately coated with a thin layer of silicon dioxide of 13 nm, 17 nm, and 30 nm thick. Initially, thicknesses of 10 nm, 20 nm, and 30 nm were planned, but this result could be achieved only after additional calibration of the deposition rate in the vacuum unit. The thickness of the deposited coating was under broadband optical control with time correction (at a known rate of substance deposition) (see also Appendix A).

### 2.3. Experiment

Using the PC, we sequentially tune the AOTF, starting with a wavelength of approximately 480 nm and ending at approximately 780 nm. As we approach the extremes of the wavelength range, the light intensity decreases. If necessary, the measuring range can be extended from 450 nm–900 nm to 900–1700 nm via proper cell driver tuning. The camera shoots at four exposures (0.1 ms, 0.5 ms, 2 ms, and 5 ms) to ensure a high dynamic range. A similar uncoated LC-7 glass prism is used as a reference of the incident radiation for calculation of the reflection coefficient *R*. As the observation takes place at angles exceeding the angle of total internal reflection, the reflection coefficient for the reference prism is close to 1. The reference prism allows us to take into account the deviations caused by the aberrations in the lenses, the insufficient uniformity of illumination, and the Fresnel reflections. To determine the angular scales in the images, we utilize a green laser to measure the angular position of the central laser spot from the reflection from the base. Then, using the rotating platform, we mark the positions of the light spot with increments of 30 angular minutes. We note that the resulting angular scale remains linear across all measurements, which indicates negligible aberrations in the lens and sufficient collimation of the beam. The measured angular scale refers to the angle of incidence of light onto the face of the prism. We recalculate it into angles of incidence onto the base of the prism, taking into account Snell’s law and the known dispersion in the glass.

## 3. Results

**Figure 2:**
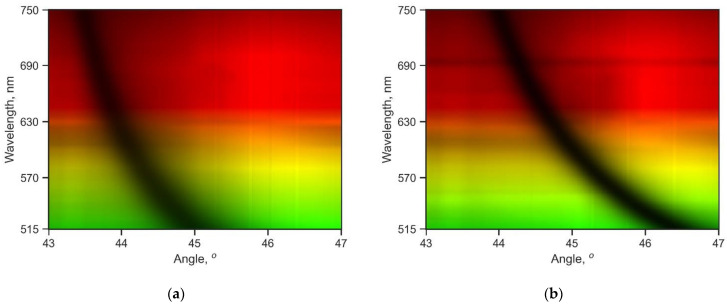

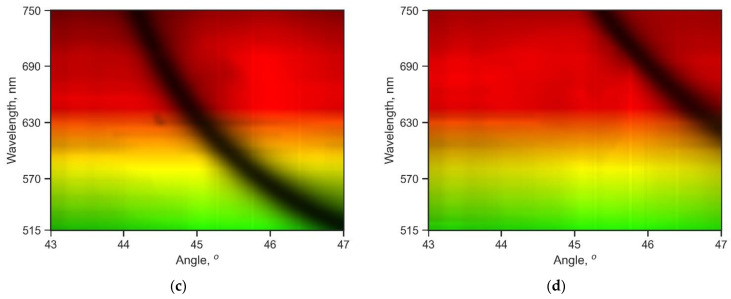
Experimental spectral images of the resonance curves (dependence of the reflection coefficient *R* on the angle of incidence *θ*) for the LC-7 glass prism with the thin 55 nm silver film and various thicknesses of the dielectric coating (silicon dioxide). The color corresponds to the real color visible to the human eye. The thickness of the dielectric coating measured by the spectrophotometer is (**a**) 0 nm, Sample 1; (**b**) 13 nm, Sample 2; (**c**) 17 nm, Sample 3; and (**d**) 30 nm, Sample 4.

The obtained experimental data in the form of a set of images from the camera and their corresponding spectra were processed with a program written in Python. The source code and images are available on Github. As the AO cell control driver was not pre-calibrated, the measurements were taken with a constant radiofrequency step of 1 MHz, starting at 62 MHz and up to 110 MHz (48 wavelength measurements). The measurement data were then recalculated and linearly interpolated to a uniform grid with wavelengths of 515 to 750 nm, as shown in Figure 2.

In Figure 2, we observe a spectrum in terms of the wavelength λ and angle *θ* of the resonance curves. This spectrum clearly repeats in shape the known dispersion curves ω (*k*), as the wavelength is inversely proportional to the frequency ω and the wave vector *k*, and *k* of the surface plasmons is proportional to the angle of incidence *θ*. Figure 2 is inspired by Figure 2 in [48], where SP dispersion curves for the flat and textured metal surfaces are shown, obtained by the modified prism coupling method using a photographic film.

**Figure 3:**
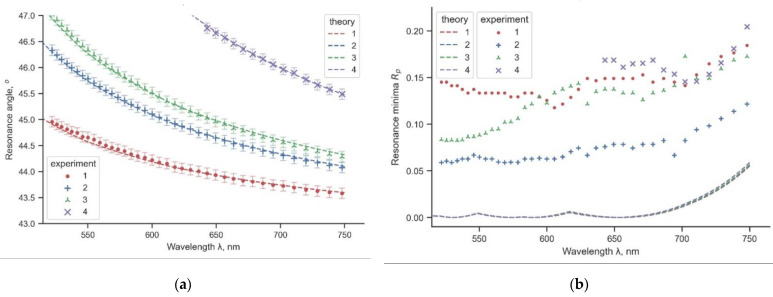
Experimental (solid) and simulated (dashed) resonance curves for different samples: 1—without a dielectric coating; 2, 3, and 4—with sequentially increasing coating thickness (see Table 1). The wide semi-transparent line shows the confidence interval: (**a**) dependence of the resonance angle on the wavelength; (**b**) dependence of the minimum of the reflection coefficient on the wavelength.

Having the known optical constants for silver (e.g., from refractiveindex.info [49]), we found that the experimental curves for the resonance angles in Figure 3a (value of the angle *θ* at the minimum of the reflection coefficient *R*) agree well with the theoretical values. We calculated the reflection coefficient *R* for the multilayer structure by the transfer matrix method [50,51]. However, the values of the reflection coefficient *R* in Figure 3b differ. The larger values for the reflection minimum can be explained by the fact that the calculation was performed for a monochromatic light, but the light after the acousto-optic cell has a wider spectral width, which leads to a decrease in the contrast of the resonance curve. 

**Figure A1:**
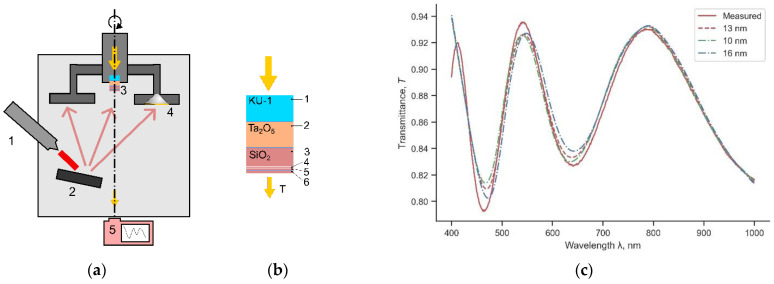
(**a**) The schematic of the thin-film sputtering chamber and optical control unit: ion gun (1) irradiates target (2); the dispersed substance is deposited on control sample (3) and SPR prism (4) fixed on the rotating carousel. The sputtering thickness is monitored with a spectrometer (5). (**b**) The final layout of the sprayed layers on the control sample: 1—KU-1 glass; 2—pre-sprayed Ta_2_O_5_ layer; 3—pre-sprayed SiO_2_ layer; 4, 5, and 6—SiO_2_ coatings. (**c**) An example of the measured transmission spectrum of the control sample for coating thickness (red solid line) and a model approximation for 13 nm coating thickness (dashed line), and model approximations within a confidence interval of 10–16 nm (dot-dashed lines).

From Table 1, we see a difference in the dielectric coating thickness measurements. However, the measured values fall within the confidence intervals. We should note that the accuracy of the spectrophotometric control applied in the thin film production is not the best in its class and is given only for reference purposes. In our case, the SPR spectroscopy is a more precise and convenient technique because, at a well-defined resonance dip, a shift in the position of the resonance angles at multiple wavelengths is easier to interpret than a shift in the spectral maxima and minima of the gradual spectral curve obtained by spectrophotometry (compare Figure 3a and Figure A1c). Besides that, SPR spectroscopy allows measuring the dielectric thickness directly on the sample itself, whereas spectrophotometry measurements are performed indirectly on a specially prepared control sample (see Appendix A).

## 4. Discussion

In the presented experimental setup, we used the AOTF produced by the Scientific and Technological Centre of Unique Instrumentation of the Russian Academy of Sciences. The AOTF combines the functions of a monochromator and a light polarizer; the AO crystal used is transparent in the visible and infrared ranges, and its optical design is suitable for imaging purposes, allowing parallel angular and spectral SPR measurements. This makes the AOTF an ideal tool for miniaturizing an SPR spectroscopy setup and allows using it for the development of an AOTF-based SPR videospectrometer.

The determination of angular coordinates in this experiment has a high error of 10’. However, the precision can be improved up to 1’, which is the limiting precision of the nonius in the rotating platform. This improvement would lead to a more accurate determination of the thickness of the thin film, up to 1 Å. To achieve this, it is necessary to reduce the influence of the error sources and increase the sensitivity of measurements.

Several sources of errors can be listed:

− chromatism in optical elements, that is, in the AOTF and lenses;
− misalignments in the setup;
− inaccuracy in the initial assumptions of the optical model.

*Chromatism.* The angular error introduced by the chromatic dispersion of light in the AOTF was compensated for to some extent by the use of the glass wedge. We can further improve the accuracy by calibrating the angular scale of the camera at different wavelengths instead of just one. Additionally, we can use additional calibration lasers of different wavelengths (e.g., the green KTP laser (λ = 532 nm) and red-diode-pumped semiconductor laser (λ = 671 nm)) to ensure coincidence of the resonant angle positions in both coherent and noncoherent light. The chromatism caused by the collecting lens in front of the prism can also be eliminated via application of a second off-axis parabolic mirror.

*Misalignments.* Another source of error in this experiment is the placement of the prism on the 3D-printed holder. This allows accurate positioning of the prism relative to the rotating platform. However, owing to the imperfection of the 3D printing (accuracy of about 0.4 mm), the holder plane is slightly inclined relative to the optical table, which results in a slightly inclined SPR line, with the bottom of the line deviating from the top by about 2’. This error can be eliminated via the introduction of a tilt platform.

*Inaccurate optical model.* Another factor not taken into account in these measurements is the roughness of the metal and dielectric films. The optical model used assumes the existence of sharp boundaries between the metal, dielectric, and air layers. However, if the surface is rough, then, instead of a sharp boundary between the layers, there will be a boundary with a gradient change in the refractive index. The roughness of the silver film can be estimated at 2–3 nm [52]. We can take this into account by adding gradient-index transition layers at the metal–dielectric and dielectric–air interfaces. The optical properties of these layers can be described with an effective medium approximation, e.g., the Maxwell–Garnett model [53].

To increase the sensitivity of measurements, it is better to recalibrate the AOTF for a shorter wavelength range, down to 400 nm, because, at shorter wavelengths, the resonance angle deviates by larger values. Additionally, to improve the determination of the reflection coefficient *R*, one could increase the light output via compact assembling of the optical setup. Currently, the light from the AOTF travels a distance of about 1 m to the prism, which results in a relatively low intensity compared with the brightness of the light exiting the AOTF.

**Figure 4:**
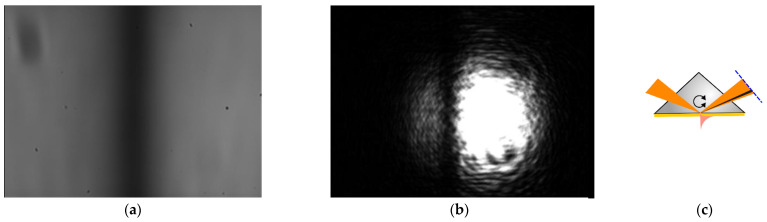
Comparison of images of the SPR curve with a monochrome camera: (**a**) for an AO filter with the central wavelength λ ≈ 633 nm; (**b**) for a helium-neon laser, λ = 632.8 nm. The images are the angular distribution of the reflected radiation from the base of the prism; (**c**) SPR observation design (top view); the dashed line is the image plane.

Despite the experimental errors listed above, it is known that SPR spectroscopy is more sensitive than spectroreflectometry [54]. Besides, the use of the AOTF allows obtaining less noisy images, because there is no parasitic interference, which can be seen from the comparison of coherent and incoherent monochromatic light sources in Figure 4.

However, the AOTF has the disadvantage of its light spectrum being broader than that of the laser. This leads to a slight decrease in the contrast of the overall picture. In addition, the focusing spot of the AO light on the surface of a thin film is an order of magnitude larger than the laser spot. Thus, the laser source has a higher lateral resolution in the case of surface microscopy [55]. However, for non-destructive testing, a wide spot can be an advantage, as it excludes the accidental influence of microscopic defects, such as pinholes, known as “starry sky”, or settled dust particles.

Furthermore, noise can be seen on the resonance minimum plot *R_min_* in Figure 3b. However, the data from Figure 3b were not used in the determination of the film thickness. Meanwhile, an analysis of Figure 3b shows that the SPR efficiency varies with different dielectric coating thicknesses, which contradicts our expectations. According to the calculations, the depth of the resonance curve (i.e., the value of *R_min_*) shall not change, as seen for the dashed lines in Figure 3b. The depth of the SPR curve depends on the thickness of the metal film. If diffusion of the dielectric into the metal during the sputtering is assumed, we can see that the metal film gradually decreases in thickness, which leads to conditions closer to optimal SPR at the 9 nm coating (line 2 in Figure 3b). It is also noticeable that the corresponding resonance curve (the thick black line in Figure 2) has the most pronounced profile. However, as almost all experimental lines lie in the error interval, we cannot insist on the correctness of our hypothesis. Meanwhile, the results of the measurements of thicker coatings are not presented in this article; the downward trend in the SPR efficiency persists. Further investigation into the issue of metal diffusion into the dielectric is needed, but our approach to SPR spectroscopy can offer a promising way to examine the transient diffusion layers, which are important in understanding of the corrosion processes of thin metal films.

The high accuracy of the SPR technique in combination with spectroscopy will make it possible to study ultrathin films and, in particular, gradient-index films. The physical reason for the ability of each wavelength of light to provide information about the inhomogeneities of the film thickness, in addition to the dispersion of its optical constants, is the nonlinear interaction of surface electromagnetic waves with the substance of the film. The energy of the field is distributed exponentially with respect to the normal surface, allowing for subwavelength characterization of the film structure. This is important, for example, because graded-index thin films enable the achievement of high laser stability of mirrors [56], change in the mechanical properties of films, increase in the efficiency of scintillation detector BGO crystals [57], and so on. However, their manufacture requires precise non-destructive testing. The common X-ray diffraction analysis is difficult to use continuously in production and requires an ultra-smooth surface, and traditional spectrophotometry does not provide the necessary accuracy, as the strength of the interaction of light with ultrathin layers is too small [58]. SPR spectroscopy using compact AOTFs makes it possible to bring real-time optical control into existing thin film deposition facilities.

## 5. Conclusions

We obtained combined spectral and angular surface plasmon resonance curves and observed the expected difference in the SPR spectra for slightly different coating thicknesses. The image of the SPR curve obtained with the AOTF-filtered noncoherent light source is less noisy compared with the laser image. This promising result shows the potential of using the AOTF for SPR spectroscopy in the angular interrogation mode and for hyperspectral SPR imaging for sensorics and non-destructive testing applications, in not only the visible, but also the IR and THz ranges.

## Figures and Tables

**Table 1:** Measured thickness values in nm for the dielectric coating.

Method	Sample 1	Sample 2	Sample 3	Sample 4
Spectrophotometry	0	13.0 ± 3.0	17.1 ± 4.2	30.0 ± 5.2
SPR spectroscopy	0	8.7 ± 1.5	12.0 ± 1.4	25.9 ± 1.4

## Data Availability

The source code is publicly available at the author’s Github repository: https://github.com/KhasanovISh/Public. The data presented in this study are openly available in FigShare at 10.6084/m9.figshare.22015910.

## Appendix A. Method of Determination of the Thickness of the Deposited Layer Using a Spectrophotometer

For measurements of the thin layer thickness, we used a control sample of high-purity fused silica glass KU-1 ($n_{D}=1.4584$, $n_{e}=1.4601$) with a diameter of 22 mm and thickness of 6 mm, with a pre-sprayed multi-layer dielectric coating (two layers: Ta_2_O_5_ with a thickness of 185 nm and SiO_2_ with a thickness of 264 nm). This coating was applied in two steps: tantalum oxide deposition (working pressure of 2.8 × 10^−2^ Pa) and silicon dioxide deposition (working pressure of 1.75 × 10^−2^ Pa). The sputtering of coatings was carried out by ion-beam at an initial pressure in the chamber not worse than 8 × 10^−4^ Pa. After each step, the transmittance of the sample was measured with the Cary5000 spectrophotometer in the wavelength range of 400–1100 nm. Pre-calibration (normalization) of the signal to zero and 100% transmittance was performed. Only one control sample was used in all spectrophotometry measurements, so the dielectric coatings were sprayed sequentially one on another (see Figure A1b), which led to an increase in the measurement error of each successive layer (Table 1). From the data obtained, the thicknesses of the layers of tantalum oxide and silicon dioxide were determined with the help of a program written in Python. During the sputtering process, the desired thickness is obtained by fitting theoretical to experimental spectra. The estimated error of the thickness measurements was found via Monte Carlo simulation on synthetic data sets (M = 100) from the spectral transmittance points (N = 700) [59].

The measurement error with Cary5000 was minor, about 0.1% for a sample after four measurements with a shift to take into account the uneven coating thickness. The sputtering error due to variations in the position of the parts on different tooling was 1%. This error was calculated from measurements taken on different holders for the same process. The error in measuring the transmittance spectrum during the sputtering process and in the control sample was influenced by the stability of the optical signal, which was disrupted by the vibration from the rotation of the carousel. The control sample and prism were located in different positions in the chamber (see Figure A1a), which could result in a slight difference in their coating thicknesses.

For calculation of the thickness of the sputtered layer, OptiLayer 7.68 software was used for the determination of the refractive index through the transmission of a thick (over 200 nm) layer of silica sputtered on a quartz substrate over a known layer of tantalum oxide. The refractive index dispersion for each dielectric used in the sputtering process must be measured beforehand. These values may vary slightly from one process to another, which complicates the film fabrication process. The main source of error in the determination of the silicon oxide thickness was the measurement of the refractive index used in the model for a sputtering process with a higher oxygen concentration. In our case, during the sputtering onto the SPR prism no supplemental oxygen flux was used in order to prevent oxidation of the metal film on the prism. This led to a change in the oxygen concentration. As a result, the model and measured spectra do not fit well (see Figure A1c).
